# Gut instinct drives discovery: an interview with Hans Clevers

**DOI:** 10.1242/dmm.052860

**Published:** 2026-03-19

**Authors:** Hans Clevers

**Affiliations:** Oncode Institute, Hubrecht Institute, Royal Netherlands Academy of Arts and Sciences and University Medical Centre, Uppsalalaan 8, 3584 Utrecht, The Netherlands


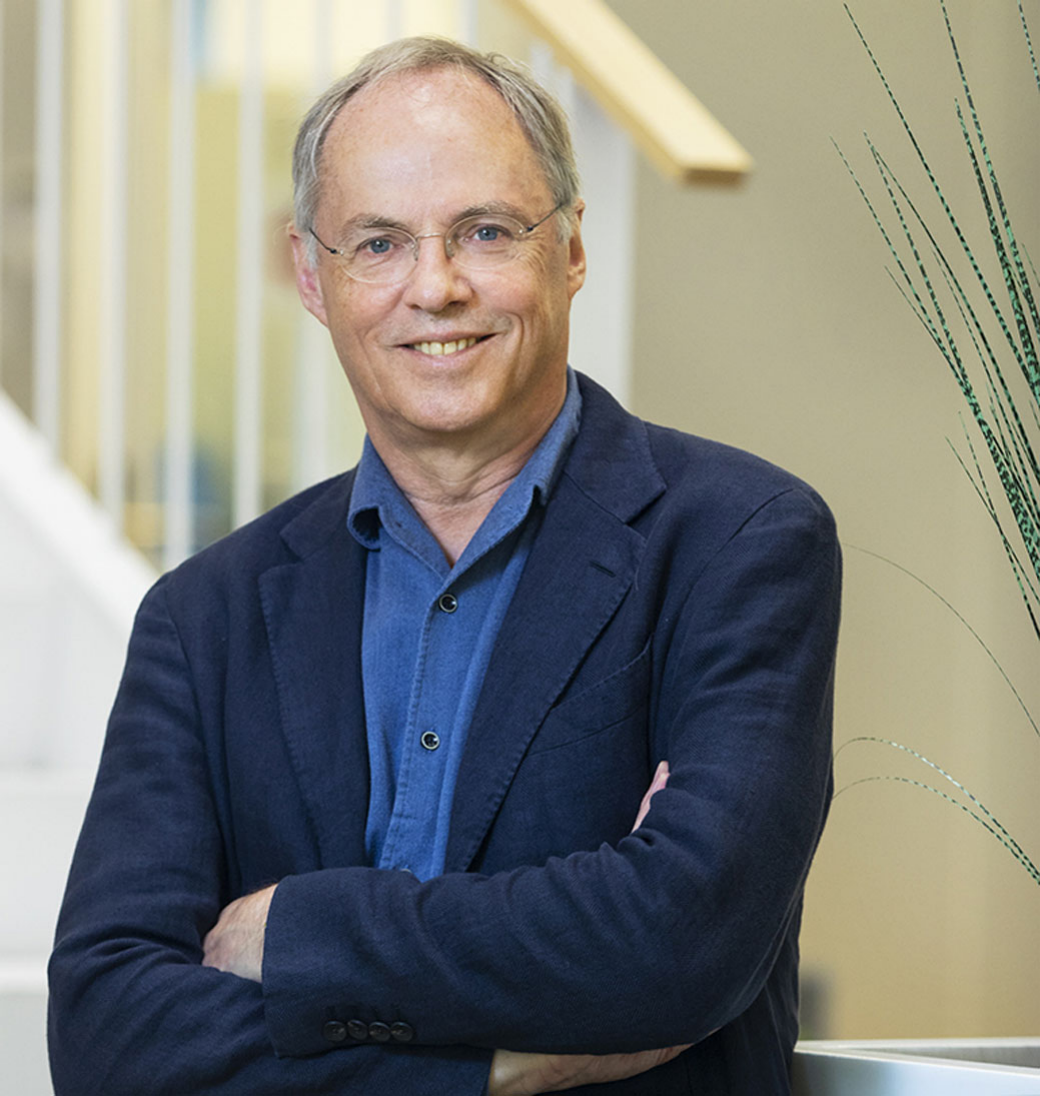


**Hans Clevers**

In 2009, Hans Clevers discovered how to mimic 3D human tissue *in vitro* in the form of intestinal organoids (Sato et al., 2009). Since then, the organoid repertoire has been expanded, refined and used extensively to study disease biology and for drug development. His journey toward this discovery began at the University of Utrecht where he completed his undergraduate degrees in biology and medicine. Opting for a career in research over medicine, he did his postdoc in Cox Terhorst's Lab at the Dana-Farber Cancer Institute, Boston, then returned to the University of Utrecht to set up his own lab in 1989. In 2002, he became the director of the Hubrecht Institute in Utrecht. Throughout his academic career Hans has founded spin-out companies and has advised biotech and pharma companies. Then in 2022, he took on a more established role as Head of Pharmaceutical Research and Early Development (pRED) at Roche. He has now stepped back into his academic labs in Utrecht. Owing to his strong leadership in the field of stem cell research, Hans served as International Society for Stem Cell Research (ISSCR) president in 2017. He has also won many prestigious awards for his cutting-edge research, most notably the Breakthrough Prize in Life Sciences in 2013, for the discovery of organoids that stemmed from years of research into Wnt signalling in the gut. Here, we discuss his career transitions throughout the years, how academia and pharma can synergistically drive drug discovery and the future of organoid and stem cell research.

**How did your medical training influence your career as a scientist?**

I always knew that I was going to be a scientist. When I was 18, I went to university in Utrecht to study biology. This was 1975, and biology was not very exciting then: it was dominated by anatomy, taxonomy, Latin names – very little mechanism and very few tools to do really informative experiments. I was disappointed. In Utrecht, we had our lectures in 19th-century buildings, with poor heating in the winter and certainly no glamour. So, I enrolled in medical school as well and, in the end, I completed both degrees.

Around that time, biology gradually became much more interesting. Recombinant DNA technology was being introduced, and monoclonal antibodies were invented. I eventually decided not to practice medicine but to become a scientist, a decision I've never regretted.

Looking back, my medical training has been very useful for what we do now. For example, when we worked on Hashimoto's disease, it helped that I already understood the condition and, more broadly, what the real challenges in the clinic are. Sometimes basic scientists work on what they think are clinical problems, but they're not the ones clinicians actually face.

Having an MD also opens doors. I'm sure it helped me become a professor, and it certainly helps when I walk into a doctor's office to discuss collaborations because I know the language they speak.

**How did you decide to move into your role at Roche and how was the transition?**

Ever since my postdoc in Boston, I've had some involvement with industry, but my academic lab has always been my anchor. Many people who move into administrative roles: department head or dean, end up leaving their own science behind. I've always felt that to be an effective administrator, manager or leader, you need to keep one foot in the core business of the organisation. In a scientific institute, that core business is science, so I've always tried to maintain my own research lab.

Over the years I advised several biotech companies and founded a few myself, but always on the side of my academic work, never as my main focus. About seven years ago I was invited to join the Supervisory Board of Roche, which sits above the executive board that includes the CEO and CSO. We met six to eight times a year. The board typically includes a number of CEOs from other major companies, two members of the founding family – Roche is still family owned – and two scientists.

I was pleasantly surprised by the culture. There was, in many ways, more mutual respect in that boardroom than I've often seen in academia, with its anonymous peer review and sometimes harsh criticism. I also began to appreciate just how challenging drug development is: it's a long, expensive and incredibly complex process that involves many disciplines. It's almost a miracle that it sometimes works at all: no more than 1% of projects ultimately lead to a drug that benefits patients. I was fascinated by that complexity and scale. Yet, I realised that, although the scale is very different, the core challenges are not so different from academia: working with people, building teams, fostering collaboration, setting a strategy but staying flexible.

I served on the Supervisory Board while still running my lab. My plan was to focus on my research for as long as I could think clearly and not take on any major new roles. But then a Head of R&D position at Roche opened up. We decided to move to Basel, and that role turned out to be more than a full-time job. I was able to negotiate an arrangement with my employers in the Netherlands that allowed me to remain as an advisor to my two labs in Utrecht, carefully avoiding any conflict of interest. The two labs together comprise about 40 PhD students, postdocs and technicians. So, for almost four years I was effectively doing two jobs, which was intense yet fascinating.

I also had to learn how to lead an organisation of thousands of people rather than dozens or a few hundred, which is quite a different challenge. And, as part of the executive board, I had to engage with geopolitical changes and global uncertainties, which were not insignificant in recent years. I actually found this addictive – intellectually and professionally it was extremely interesting. I had initially committed to three years; in the end I stayed close to four. I'm 68 now, and for the rest of my working life I plan to concentrate on my labs.

Meanwhile, we have been building a new institute within Roche called the Institute of Human Biology. Roche has a strong history in this space: it once supported the Basel Institute of Immunology, a world-renowned centre where three Nobel laureates worked in the 1970s, 80s and 90s. That institute closed around the year 2000. The new institute is different in structure – it is formally part of the company – but the idea is similar: it functions essentially as an academic institute embedded within Roche.

A key principle is that the junior and senior PIs at the Institute of Human Biology do not work directly on Roche's ‘portfolio’ programmes, i.e. the specific compounds that have been selected as drug candidates. Once a compound enters formal development, the investment is so large that the organisation becomes understandably risk-averse; you do not hand such a molecule to someone who might, through an exploratory experiment, generate data that imperil the programme. Instead, the institute is designed to act as a bridge between academia and Roche. Its investigators can collaborate more freely with external groups and with scientists in Roche's pRED organisation. In that role, they focus on identifying new disease targets, developing novel *in vitro* assays and supporting pharmacology.

This has become highly topical. The European Medicines Agency (EMA) has long encouraged the search for alternatives to animal experimentation, but in the last two or three years the FDA has dramatically accelerated this shift. The latest guidance appears to indicate that, within about five years, for large molecules such as antibodies, animal data will have to be largely replaced by New Approach Methodologies (NAM) in drug development, which refers to non-animal, human-relevant testing methods, including *in vitro* assays (organoids, organ-on-a-chip technology) and computational models. This means that, over the next few years, we must establish, validate and robustly scale up a large number of *in vitro* assays. The new institute is intended to help drive this transformation, which makes it particularly timely. It will soon move into a new purpose-built facility in Basel, right at the heart of Roche's headquarters.

**How do you decide when a research project or technology is mature enough to progress into a new biotech company or into pharma?**

The ‘business plan’ of an academic lab is essentially this: you win grants, hire people, build a team; and the product is a discovery that becomes a paper, perhaps identifying a new disease target, a new mechanism or a new assay. What I've learned, especially from seeing the pharma side, is that there is a huge gap between what academia delivers in the form of a nice paper and what pharma can actually use.

Biotechs are very good at bridging that gap, particularly when they are founded by the original inventors. Looking back, I think I – and most academics – are quite naïve about what constitutes a good starting point for a biotech. Venture capitalists tend to have more experience judging that. However, pharma scientists are often quite conservative; they can be slow to recognise truly novel ideas and are generally more risk-averse. I would say that 99% of genuinely new targets and mechanisms originate in academia, not industry.

This transition from academia, where the real discoveries are made, via biotech with people who have the courage and maybe the naivety to pursue a new idea, works well.

This transition from academia, where the real discoveries are made, via biotech with people who have the courage and maybe the naivety to pursue a new idea, works well. From an academic perspective, biotech budgets look huge; from a pharma perspective, they are actually small. That makes biotech a relatively inexpensive way to find out whether something is a brilliant idea or a bad one. You get a few years to test it. Pharma is also much more comfortable evaluating a project that has been developed in a biotech than one that's still in an academic lab.

As for when it's a ‘good idea’ to make that leap: sometimes the craziest ideas turn out to be fantastic. Let me give you one example I probably would not have bet heavily on myself. My lab discovered that LGR5 is a marker for stem cells (Barker et al., 2007). One of my best friends, Ton Logtenberg – whom I've known for about 50 years (we used to be colleagues in immunology and we still go skiing together every year), started a company called Merus. Between us we came up with the idea of building a library of biospecific antibodies: one arm recognising stem-cell markers, such as LGR5, the other recognising members of the EGF-receptor family. Merus created a library of roughly 500 antibodies; together we screened them and found one that performed spectacularly well.

That project began with an EU grant involving Ton's then-small company and my former postdoc Eduard Batlle, who now is in Barcelona. My lab's contribution was quite a few years ago but the antibody is now in phase III cancer trials. Merus was recently sold for a substantial sum to Genmab, also in Utrecht. The molecule appears to be very safe and clearly superior to existing options.

What's especially interesting is that it was developed entirely by using organoids – no animal models, no traditional cell lines. The key was that we used ‘normal’ and tumour organoids from the same patients and screened for molecules that killed the cancer organoids but spared the normal ones. That had never really been done before. So-called ‘normal’ cell lines, if you transplant them, will form cancers, whereas normal organoids are genuinely normal. By using this approach, we identified molecules that are truly selective, which addresses the central business challenge in oncology: finding something that kills cancer cells but leaves normal cells alone. In fact, most existing cancer drugs will kill normal cells at least as well as they kill cancer cells in our organoid assays.

In the end, discovery science remains a game of gambles and odds. In my lab we try to run through hundreds of ideas quickly, with pilot experiments that are just good enough to tell us whether there is something there. The biotech world does the same at a larger scale: you generate many ideas – some crazy, some less so – and you test them. Most will fail, and the corresponding companies will disappear. But when it works, the result can be spectacular.

**Since generating the ‘mini-gut’ your lab has set up organoid systems for a wide range of tissues that are used for various areas of research. How do you keep up with all these different fields?**

First of all, probably half of the projects in my lab start without consulting me. There is a lot of freedom in the group, as long as people stay within some clear guardrails. The lab is fundamentally strong in technology. Once you have a powerful platform you can plug in many different biological models.

In practice, when a new postdoc joins, we sit down and I ask which tissue or disease area they are most excited about. Then we do a lot of reading together. I'm also guided by insights in diseases that fascinated me during my medical training. Very often we bring in an established expert in that particular field as a collaborator – both to help us avoid obvious mistakes and to teach us the ‘culture’ of the field. Every area has its own terminology, its own standard controls, its own trusted reagents.

Typically, we publish a few papers together with the expert collaborator and then hand the project over to their lab. That's why you may see a breast-cancer paper from us, but the follow-up work usually comes from our partners, not from us. This model works well. Without collaborators, it would be very hard: a field is unlikely to welcome an outsider, who suddenly claims to have solved a problem they've worked on for years or to tell them that a long-held belief is wrong.

We now know that there is probably no general system for how tissues use their stem cells.

Yet, sometimes, that outsider perspective is exactly what allows one to challenge dogma. You come in from a different angle, with a fresh open mind, and you question things that insiders have stopped questioning. We experienced this with LGR5-positive gut cells. Because they are abundant and divide every day, most people were convinced they could not be stem cells. Textbooks said stem cells divide slowly. The bar was, therefore, very high for us to prove that, despite not fitting the textbook definition, these cells are, in fact, stem cells. We now know that there is probably no general system for how tissues use their stem cells. For instance, the liver does not appear to rely on classic stem cells at all and recruits its mature cell types as reserve stem cells when it is damaged.

**What is the next innovative use of organoids and how can they be advanced further?**

My personal opinion is that the strength of the organoids is their simplicity.

First of all, as experimental tools in biology and biomedicine, organoids are now well established. If you look at the number of papers that use or cite organoids, it still increases every year. Recently, there has been a lot of discussion in one particular direction: some people argue that we should make the models more complex by adding microbiota, nerves or immune cells. My personal opinion is that the strength of the organoids is their simplicity. They are more than a cell line. They really are primary cells, the way we grow them. When we make gut organoids, for example, they consist purely of gut epithelium. That means the experiments are very clean and highly informative. You can see immediately whether there is an effect or not; often you don't even need statistics.

Of course, many scientists point out that there is no stroma or immune system, so they start adding components. But as soon as you do that, you lose the advantage of simplicity, and the added complexity quickly introduces experimental noise. Organoids will never become a complete organ, let alone a whole animal or human being. There is also a lot of enthusiasm for ‘multi-organ’ systems – putting three or four organoids (gut, liver, brain, etc.) on one chip. It sounds very attractive but, once you try it, you realise how much complexity you create, as you do not really know how to connect gut to liver or liver to brain in a meaningful and standardised way.

So, my recommendation is to keep organoids simple. If you want to add an immune component, add one defined T-cell population. Instead of adding a whole microbiome, add a single microbial species and then interpret or ‘translate’ that back to the real situation. That's essentially what we did for genotoxic *E. coli*: start with a minimal, well-controlled system and build understanding from there (Pleguezuelos-Manzano et al., 2020).

Looking ahead, I think that for organoids to be used more broadly beyond academia, the key will be automation, standardisation, scale up and rigorous validation. If we believe an organoid assay can detect toxicity better than animal experiments, we must demonstrate this convincingly and validate it. My sense is that a lot of work in the coming years will focus on taking these basic models, perhaps adding just one extra element – one microbial species, one immune cell type, one nerve cell type – and then automating, miniaturising, validating and making them robust. That is probably where the field is going.

**Having previously served as the ISSCR president, from that broader perspective, what are the next big challenges in stem cell research?**

The ISSCR has been very effective at insisting on high-quality science. For example, it has consistently and openly challenged areas like the mesenchymal stem cell field, where many of us never believed these cells met the definition of ‘stem cells’, despite their enormous clinical popularity and the large number of ongoing trials. The ISSCR has also taken a strong stance against commercial ventures offering unproven and, often, dangerous ‘stem cell’ therapies that lack any real scientific foundation.

My own view – and one shared by the ISSCR – was that stem cells would transform transplantation medicine: either by replacing organ transplants through cell or organoid therapies, or even by enabling us to grow transplantable organs. In reality, progress has been extremely slow. Bone marrow transplantation is routine worldwide, and there are some successes with skin and corneal grafts grown from cultured stem cells; but, beyond that, very little has reached the clinic, despite intense efforts.

Within the ISSCR we, therefore, had a serious discussion about a strategic shift: using what we've learned from stem cells less for direct cell therapy but more to build *in vitro* human models for drug development and to understand disease mechanisms. For the first time, we can culture parts of the human brain or retina, and study human biology and pathology in ways that were previously impossible.

The ISSCR has been pursuing to bring more clinicians into the cell-therapy space because you need doctors to run trials, which worked to engage industry as well. But, beyond CAR-T cells, industry has been rather cautious about cell therapies: they are logistically complex, ethically sensitive, and raise difficult questions about tissue ownership and how (or whether) you can build a sustainable business model, for example, around a transplanted kidney. By contrast, companies are very interested in using stem cell-derived and organoid-based models for drug discovery and development, where these technologies can help produce safer more-effective drugs more quickly and at lower cost.

**You've made seminal discoveries in your career, but what discovery or research project have you found most exciting to work on?**

If I had to give an overview of my research, it might look like a straight line of successes. When I received the Breakthrough Prize in 2013 – with what felt like an embarrassingly large amount of money – I invited everyone who had ever worked in my lab to Amsterdam for a long weekend that included a symposium in Amsterdam's science museum NEMO. Many of my colleagues went on stage to say a few words about their time in the lab and what they were doing afterwards. To my surprise, almost all of them began by talking about the projects that had failed – projects I had more or less forgotten.

I had always had the impression that almost everything we tried had worked. Looking back now, I realise that what I remember as a smooth, linear progression was, actually, built on countless dead ends. The real process was to try many things, most of which do not go anywhere, and then choose the few promising directions for the next step.

There are a few highlights I remember clearly. One was identifying the role of TCFs in the Wnt pathway (Molenaar et al., 1996). That led us to show that Wnt signalling drives stem cells and cancer in the gut (Korinek et al., 1997; Morin et al., 1997). About ten years later, in 2007, we used that insight to identify the gut stem cells themselves (Barker et al., 2007). With those stem cells in hand, we were then able to develop organoid technology (Sato et al., 2009). Those four projects form the visible milestones. But they sit on top of many other projects that never worked out; and that, in reality, is what the ‘single line of successes’ was built from.

**What do you enjoy doing outside of work?**

Skiing, sports, reading books. I used to read two books a week. But no longer, the past four years have just been too busy. I'm looking forward to doing that again. And generally socialising with family, friends and people in general.
